# Application of artificial intelligence in endoscopic gastrointestinal tumors

**DOI:** 10.3389/fonc.2023.1239788

**Published:** 2023-12-06

**Authors:** Yiping Xin, Qi Zhang, Xinyuan Liu, Bingqing Li, Tao Mao, Xiaoyu Li

**Affiliations:** Department of Gastroenterology, The Affiliated Hospital of Qingdao University, Qingdao, China

**Keywords:** artificial intelligence, deep learning, gastric cancer, colorectal cancer, adenoma detection rate

## Abstract

With an increasing number of patients with gastrointestinal cancer, effective and accurate early diagnostic clinical tools are required provide better health care for patients with gastrointestinal cancer. Recent studies have shown that artificial intelligence (AI) plays an important role in the diagnosis and treatment of patients with gastrointestinal tumors, which not only improves the efficiency of early tumor screening, but also significantly improves the survival rate of patients after treatment. With the aid of efficient learning and judgment abilities of AI, endoscopists can improve the accuracy of diagnosis and treatment through endoscopy and avoid incorrect descriptions or judgments of gastrointestinal lesions. The present article provides an overview of the application status of various artificial intelligence in gastric and colorectal cancers in recent years, and the direction of future research and clinical practice is clarified from a clinical perspective to provide a comprehensive theoretical basis for AI as a promising diagnostic and therapeutic tool for gastrointestinal cancer

## Introduction

**Figure 1:**
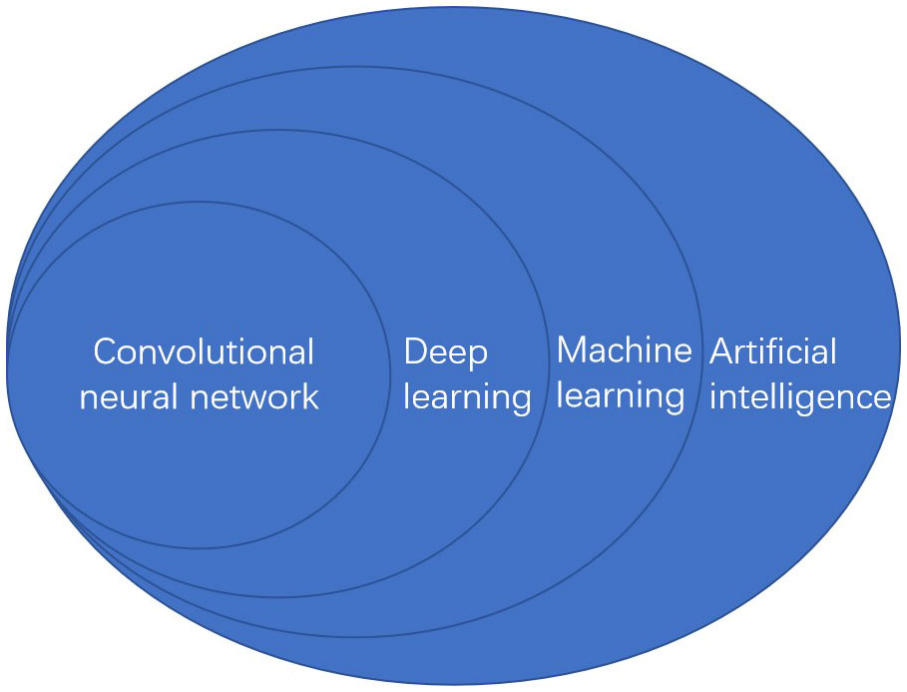
The relationship of artificial intelligence classification.

Recently, artificial intelligence(AI) technology has been successfully adopted in health care diagnostics, which is the branch of computer science (1). It is used to attempt to learn and solve problems by emulating human-like mind and cognition (2).Machine learning (ML) and deep learning(DL) can be considered subsets of AI. ML-based approaches refer to the scientific studies of algorithms and statistical models that can perform complex tasks after manually extracting features (3). The algorithm can learn independently using multiple datasets without explicit instructions, which is at the forefront of AI and data science (4). In this statistical method of fitting models to data, the models are trained and learned using databases to make predictions based on new data (5). DL is a particular ML approach that developed through the advancement of artificial neural networks (ANN) and specialized in deep neural networks. A classification and recognition system for focal images can be constructed without complex image-processing algorithm (6).The algorithm can also learn and utilize interaction factors between data inputs to predict its target (4). Convolutional neural network (CNN) is the main DL algorithm used for image recognition and image processing (7, 8). After identifying and extracting specific features from the original images, the CNN uses mathematical convolution operations to perform endoscopic diagnosis, which has been reported as a successful image classification computing system (9, 10).(**Figure 1**).

In recent years, the image recognition ability of AI has become increasingly advanced (7), and DL has been widely applied in diagnostic imaging in various medical fields (11–13). Compared to traditional ANN, the DL algorithm significantly enhances the width and depth of the network. It consists of digitized inputs that can extract information from shallow, intermediate, and deep layers of images, and output layer, which is used for classification and processing lesion images at the backend (2, 14). Deep learning architecture has high detection, classification and segmentation capabilities. Therefore, it is particularly suitable for image quantization. With adequate learning, clinicians can achieve high accuracy and rapidity of AI when assessing gastrointestinal tumors, thereby improving clinical efficiency and reducing costs for patients and clinical teams (15).

## Gastric cancer

Gastric cancer (GC) remains an important cancer worldwide and was responsible for over one million new cases in 2020 and an estimated 769,000 deaths, ranking fifth in incidence and fourth in mortality globally of all cancers (16). Statistically, the relative 5-year survival rate of patients with GC is <40% (17, 18), which is attributed to the late onset of symptoms and delayed diagnosis (19). Early gastric cancer (EGC) has a high endoscopic cure rate and the 5-year survival rate exceeds 90% (20, 21). Therefore, timely and accurate diagnosis of EGC through endoscopy is a key strategy for improving survival rates. The AI-based diagnosis system has high diagnostic accuracy, which can monitor and distinguish cancer from non-neoplastic lesions in a timely manner and predict the invasion depth through gastroscopy images. The application of AI in endoscopic gastric cancer is shown in **Table 1**.

**Table 1:** The application of AI in endoscopic gastric cancer.

Authors(year)	Study design	Aim	Data		Results of AI
			Training set	Validation set	
Hirasawa et al.(2018) (22)	Retrospective	Detection of GC	13,584 images	2296 images with 77 GC lesions	Sensitivity:92.2%,PPV:30.6%
Wu et al.(2019) (23)	Retrospective	Detection of EGC without blind spots	9151 images for EGC and 24549 images for blind spots	200 images	Accuracy:92.5%,Sensitivity:94.0%,Specificity:91.0%,PPV:91.3%,NPV:93.8%
Luo et al.(2019) (24)	Retrospective and prospective	Detection of upper gastrointestinal cancer	157207 images	910598 images	Accuracy:95.5%(internal validation set),92.7%(prospective set),91.5-97.7%(external validation sets),Sensitivity:94.2%,PPV:81.4%,NPV:97.8%
Li et al.(2020) (25)	Retrospective	Detection of EGC	2088 images	341 images	Sensitivity:91.18%,Specificity:90.64%,Accuracy:90.91%
Horiuchi et al.(2020) (26)	Retrospective	Detection of EGC	2570 images	174 videos	AUC:0.8684,Accuracy:85.1%,Sensitivity:87.4%,Specificity:82.8%,PPV:83.5%,NPV:86.7%,
Ueyama et al.(2020) (27)	Retrospective	Detection of EGC	5574 images	2300 images	AUC:99%,Accuracy:98.7%,Sensitivity:98%,Specificity:100%,PPV:100%,NPV:96.8%
Hu et al.(2020) (28)	Retrospective	Detection of EGC	170 cases	125 cases	AUC:0.808 in the internal test cohort and 0.813 in the external test cohort,Accuracy:77.0%,Sensitivity:79.2%,Specificity:74.5%
Tang et al.(2022) (29)	Retrospective	Detection of EGC	13151 images	8634 images and 20 videos	AUC:0.888-0.951,Accuracy:93.2%
Horiuchi et al.(2020) (30)	Retrospective	Qualitative diagnosis of GC	2570 images	258 images	Accuracy:85.3%,Sensitivity:95.4%,Specificity:71%,PPV:82.3%,NPV:91.7%
Namikawa et al.(2020) (31)	Retrospective	Qualitative diagnosis of GC	18410 images	1459 images	Sensitivity:99.0%,Specificity:93.3%,PPV:92.5%
Kim et al.(2020) (32)	Retrospective	Qualitative diagnosis of GC	905 images	212 images	Sensitivity:83.0%,Specificity:75.5%,Accuracy:79.2%
Zhu et al.(2019) (33)	Retrospective	Prediction the invasion depth of GC	790 images	203 images	AUC:0.94,Sensitivity:76.47%,Specificity:95.56%,Accuracy:89.16%,PPV:89.66%,NPV:88.97%
Yoon et al.(2019) (34)	Retrospective	Detection of EGC and prediction the invasion depth of GC	6923 images	4616 images	Sensitivity:91.0%,Specificity:97.6% and AUC:0.981 for EGC detection,Sensitivity:79.2%,Specificity:77.8% and AUC:0.851 for prediction of tumor depth
Nagao et al.(2020) (35)	Retrospective	Prediction the invasion depth of GC	13628 images	2929 images	Sensitivity:84.4%,Specificity:99.4%,Accuracy:94.5%,PPV:98.5%,NPV:92.9%(WLI),Accuracy:94.3%(NBI),Accuracy:95.5%(Indigo)
Goto et al.(2022) (36)	Retrospective	Prediction the invasion depth of GC	500 images	200 images	Accuracy:77%,Sensitivity:76%,Specificity:78%,F1:0.662

## Detection of gastric cancer

EGC are often present in the background of gastric mucosal inflammation and are difficult to identify by endoscopists. The use of a CNN-based AI to detect GC in endoscopic images was first reported by Hirasawa et al (22).The model required a significantly shorter time for diagnosis than endoscopists and correctly diagnosed 71 of 77 gastric cancer lesions with an overall sensitivity of 92.2%, resulting in a positive predictive value(PPV) of 30.6%. Wu et al. (23)constructed a system using DCNN to detect EGC without blind spots. After validating the 200 endoscopic images, the sensitivity and specificity were comparable, and the accuracy was significantly higher. They also used it on unprocessed videos to proactively track suspicious cancerous lesions without blind spots. Luo et al. (24) developed and validated GRAIDS using a large cohort of more than one million images from different tiers of hospitals, with a diagnostic accuracy of 91.5% to 97.7%. GRAIDS can support non-expert endoscopists to a level similar to that of experts, suggesting the effectiveness of combining of AI and endoscopists. At that time, it was the largest study in the field of AI-guided cancer detection based on upper gastrointestinal endoscopic images worldwide.

Image-enhanced endoscopy (IEE) uses narrow-band spectrum or blue laser imaging to enhance micro-vessels patterns as well as color differences of gastric mucosa and structural features to improve diagnostic accuracy (37). Magnifying-IEE(M-IEE) has satisfies diagnostic ability for GC, however, its high cost of equipment and strict requirements for endoscopists limit its popularity (38).Weak magnifying-IEE(WM-IEE) has wide utility and relatively lower cost than M-IEE, providing a significant option for diagnosis of high-risk lesions (39). Since EGC shows only subtle mucosal changes, narrow-band imaging (NBI) has been reported to be a powerful tool for characterizing gastric mucosal lesions because it can use narrow light source to enhance visualization of the surface micro-vessels (40). In particular, magnifying NBI(ME-NBI) is a powerful optical technology with accurate real-time diagnostic performance in EGC. The application of CNN in ME-NBI diagnosis is a potential solution to improve the optical diagnosis (41–43). Li et al. (25) established a CNN model on 2088 images for analyzing gastric mucosal lesions observed by ME-NBI and achieved an accuracy of 90.91% in 341 still images. The diagnostic sensitivity of CNN was significantly higher than that of the experts. Similarly, Horiuchi et al. (26) verified the performance of AI for ME-NBI using 174 videos to enable real-time diagnosis of EGC and the system demonstrated an area under the curve (AUC) of 0.8684.The diagnostic performance was equivalent to or better than that of 11 endoscopic experts. A CNN computer-aided system was constructed by Ueyama et al. (27)based on ME-NBI images and achieved a high diagnostic accuracy of 98.7% among homogeneous systems and AUC of 99%. The most important difference was that the images processed by water immersion technique with maximal magnification in this study were optimal for AI-assisted diagnostics.

As the first study to evaluate AI using a multicenter validation cohort, Hu et al. (28) trained and tested a model using 1777 ME-NBI images from the database and achieved accuracies of 77% and 76% in the internal and external test cohorts, respectively. It can not only effectively improve the diagnostic performance of endoscopists of different levels, but also delineate lesion boundaries. He et al. (38) proposed a system to diagnose EGC by M-IEE and validated its effectiveness using multicenter static images from six hospitals, real-time videos, and a prospective clinical trial. This showed the great potential for the diagnosis of EGC in clinical practice. A deep CNN(DCNN) converts one level of representation into a more abstract level for prediction (7). A real-time DCNN system was developed to diagnose EGC using 21785 NBI images and 20 videos, with the largest sample size at the time (29). It showed a generalized diagnostic performance with an AUC of 0.947 on the internal validation dataset and 0.888–0.951 on the four external validation datasets. Notably, the system significantly enhanced the performance of senior (89.4%, 95% CI, 87.9–90.7%) and junior (84.9%, 95% CI, 83.4–86.3%) endoscopists. These experimental studies have promoted the development of AI technology, which has the potential for future clinical applications.

Randomized controlled studies of target population with appropriate inclusion and exclusion criteria are necessary to validate the diagnostic accuracy of AI. In addition to EGC, it is necessary to ensure the diagnosis of hard-to-detect GC, such as early undifferentiated cancers and gastritis-like cancers (44). Furthermore, most studies used DL to identify GC with WL or M-IEE, whereas few concentrated on WM-IEE (45, 46). In clinical practice, guidelines recommend the use of multimodal light sources with chromoendoscopy and white-light imaging (WLI) endoscopy, instead of a single light source (47), which underscores the importance of accurate diagnosis and risk stratification in these patients.

## Qualitative diagnosis of gastric cancer

It is sometimes difficult to distinguish benign lesions from EGC, and the PPV of biopsy using conventional endoscopy with WLI is only 3.2-5.6%. Horiuchi et al. (30) applied a CNN system to differentiate EGC from gastritis with 151 EGC and 107 gastritis images based on ME-NBI. The accuracy, sensitivity, and specificity of the system were 85.3%, 95.4%, and 71.0%, respectively. To evaluate the applicability for the classification of GC and gastric ulcer, Namikawa et al. (31) developed an AI-based system by adding 4453 gastric ulcer images to the original AI. The overall accuracies of the advanced and original AI were 95.9% and 45.9%, respectively, indicating a high level of recognition and classification. A CNN model based on ultrasound endoscopic (EUS) images distinguished gastrointestinal stromal tumors (GIST) from non-GISTs with 83.0% sensitivity, 75.5% specificity, and 79.2% accuracy (32). Therefore, it complemented the clinical practice of EUS in the diagnosis of gastric mesenchymal tumors. The application of AI to differentiate cancer from non-cancerous changes could potentially reduce the number of unnecessary biopsies.

## Prediction the depth of gastric cancer invasion

EGC refers to GC confined to the mucosa or submucosa, regardless of the presence of lymph node metastasis (48) and is classified into intramucosal cancer (T1a) and submucosal invasive cancer (T1b). Endoscopic resection has become the treatment of choice for EGC because of minimally invasive and superior cost-effectiveness (49–51). One of the most important preoperative criteria for curative endoscopic resection is tumor invasion depth. Absolute indication for endoscopic surgery is a differentiated-type adenocarcinoma without ulcerative findings (UL0), in which the invasion depth is clinically diagnosed as T1a and the diameter is ≤ 2 cm (49, 52, 53). As undifferentiated-type intramucosal adenocarcinoma of diameter < 2cm is also an absolute indication for endoscopic submucosal dissection (ESD) (54), accurate prediction of infiltration depth based on endoscopy images is a key to screen patients for endoscopic resection.

Conventional endoscopy is an effective method for T staging of EGC (55, 56), and EUS can distinguish between the different layers of the stomach wall and reveal the peri-gastric lymph nodes (57). However, influenced by endoscopists and images, EUS has no substantial effect on pretreatment T-staging of EGC patients (58, 59). Therefore, with increasing interest in the field of medical imaging, there is a requirement for a more detailed classification and higher accuracy AI system.The first investigation of the depth of GC invasion depth using a computer-aided system based on 902 images with 10-fold cross-validation method (60). The diagnostic accuracies were 77.2%, 49.1%, 51.0% and 55.3% for T1, T2, T3, and T4 stages, respectively, and the accuracy was 68.9% and 63.6% for T1a and T1b stages of EGC, respectively. Recent years, Zhu et al. (33) reported that their CNN system developed with 790 images could differentiate the depth of M, SM1, and SM2 from all GCs, with an accuracy of 89.16% and specificity of 95.56%. In a study simultaneously detected GC and invasion depth with AI, Yoon et al. (34) reported the sensitivity and specificity of tumor depth as 79.2% and 77.8%, respectively. They also analyzed factors that influence AI diagnosis, such as whether undifferentiated-type histology is correlated with low T-stage prediction accuracy. Using 16577 selected endoscopic images from different angles and distances for each lesion, the system developed by Nagao et al. (35) could diagnose invasion depth with an accuracy of 94.4%. Notably, the impact of WLI, NBI and Indigo on the ability to predict invasion depth was compared for the first time.

Zhu and Nagao reported that it is easier to diagnose the depth of invasion in advanced gastric cancer in clinical practice. Therefore, developing systems to improve the diagnostic ability for EGC would be more beneficial. Goto et al. (36) constructed an AI classifier for differentiating intramucosal and submucosal GC and devised a diagnostic method based on cooperation between AI and endoscopists. A total of test images showed that the accuracy, specificity, and F1 measure based on cooperation were 78.0%, 80.0%, and 0.776, respectively, and that the accuracy of using F1 measure was higher than that of using AI or endoscopists alone.

## Colorectal cancer

Colorectal cancer (CRC) is the third most frequently diagnosed cancer and the second most common cause of cancer-related deaths. More than 1.9 million new CRC cases and 935,000 deaths were estimated in 2020, accounting for approximately one in ten cancer cases and deaths (16). Colonoscopy plays an important role in screening and preventing CRC (61, 62). Adenomatous polyps are the most important precursor lesions and CRC usually develops from sporadic mutation-accumulating adenomatous polyps in a relatively predictable stepwise sequence (63). Colonoscopy can be used to detect and remove these lesions via polypectomy, thereby significantly reducing the incidence and mortality risk of CRC (64, 65). Evidence suggests that colonoscopy can reduce the risk of death from CRC by 67% (66) and the incidence of late-stage CRC by 70% (67).

Adenoma detection rate (ADR) is defined as the proportion of at least one histologically identified colorectal adenoma or adenocarcinoma when performing colonoscopy (68). CRCs detected after a prior colonoscopy or during the interval between surveillance colonoscopies are known as interval CRC or post-colonoscopy CRC (PCCRC). ADR is a proxy for colonoscopy quality indicator and has been inversely correlated with the risk of PCCRC (69–73). The incidence of PCCRC is estimated to be as high as 3.5 per 1,000 screened person (74). Each 1.0% increase in ADR correlated with a 3.0% decrease in the risk of PCCRC (69) and a 5% decrease in the risk of fatal interval CRC (75). It is also reported that 58% of PCCRC could be categorized as “possible missed lesion, prior examination inadequate,” which emphasized the importance of careful colonoscopy examination (76). In fact, the adenoma missing rate (AMR) of WLI colonoscopy ranges from 6% to 41% (77–79), depending on various polyps and surgical characteristics. For example, smaller polyps, flat polyps (77, 80), and left colonic location (79) may be associated with an increased AMR. The ability to examine the colorectal mucosa to the maximum extent possible and accurately identify neoplastic lesions depend mainly on the mastery of technical and cognitive skills (81). A potential solution to mitigate the variability in both endoscopic detection and histological prediction is to apply computerized image analysis to deliver computer decision-support solutions.

Moreover, accurate *in vivo* differentiation can reduce unnecessary endoscopic resections, complications, physician burden, and medical costs (82). Studies using full-spectrum colonoscopy (FUSE), which provides a 330° angle of view, showed an AMR between 7.0% (83) and 20.5% (78). AI can compensate for differences in endoscopists’ diagnostic ability due to limitations in experience, visual perception, and other human factors (84). Several computer-aided diagnostic systems have been developed and applied clinically to evaluate the benefits of improving ADR (85). The data suggest that application of CNN may lead to “resect and discard” and “detect and leave” strategies in real time, which will avoid unnecessary non-neoplastic polyp removal and improve colonoscopy efficiency and cost-effectiveness. The two significant roles of AI in CRC screening are computer-aided detection (CADe) and computer-aided diagnosis or differentiation (CADx). Using complex algorithms or CNN, CADe is used to detect lesions, whereas CADx characterizes lesions by performing optical biopsies, reducing the need for histopathological evaluation to some extent (86). Therefore, CADe can help endoscopists reduce missed polyps and augment performance, whereas CADx can interpret polyp histology more accurately (87). The application of AI in endoscopic colorectal cancer is shown in **Table 2**.

**Table 2:** The application of AI in endoscopic colorectal cancer.

Authors(year)	Study design	Algorithm type	Data		Results
			Training set	Validation set	
Misawa et al.(2018) (88)	Retrospective	CADe	411 short videos	135 short videos	Sensitivity:90.0%,Specificity:63.3%,Accuracy:76.5%
Urban et al.(2018) (89)	Retrospective	CADe with CNN	8641 hand-labeled images	20 colonoscopy videos	Sensitivity:97.0%, Accuracy:96.0%,AUC:0.991
Neumann et al. (90)	Prospective	CADe	2001 images	240 polyp videos	Sensitivity:100%,
Wang et al.(2019) (91)	Prospective	CADe	/	/	ADR of CAD 29.1% vs control 20.3%
Su et al.(2020) (92)	Prospective	CADe with DCNN	/	/	ADR of CAD 28.9% vs control 16.5%
Yamada et al.(2019) (93)	Retrospective	CADe	139961 images	4840 images	Sensitivity:97.3%, Specificity:99.0%,ROC:0.9752
Wang et al.(2020) (94)	Double-blind randomised study	CADe	/	/	ADR of CAD 34% vs control 28%
Repici et al.(2020) (95)	Randomized	CADe	2684 polyp videos	341 patients	ADR of CAD 54.8% vs control 40.4%
Repici et al.(2022) (96)	Prospective	CADe	/	/	ADR of CAD 53.3% vs control 44.5%
Wang et al.(2020) (97)	Prospective	CADe	/	/	AMR of CAD 13.89% vs routine 40.00%
Ishiyama et al.(2022) (98)	Prospective	CADe	/	/	ADR of CAD 26.4% vs control 19.9%
Soons et al. (99)	Prospective	CADe	10467 images	45 videos	PDR(polyp detection ratio):55.6%,ADR:28.9%
Chen et al.(2018) (100)	Retrospective	CADx	2157 images	284 images	Sensitivity:96.3%, Specificity:78.1%,PPV:89.6%,NPV:91.5%
Byrne et al.(2019) (101)	Retrospective	CADx	223 polyp videos	125 videos	Sensitivity:98.0%, Accuracy:94.0%,Specificity:83%,PPV:90.0%,NPV:97%
Kudo et al.(2020) (102)	Retrospective	CADx	69142 images	100 cases	Sensitivity:96.9%, Accuracy:98.0%,Specificity:100%,PPV:100.0%,NPV:94.6%(stained endocytoscopic images)
Mori et al.(2018) (103)	Prospective	CADx	61425 images	466 diminutive polyps	NPV:93.7%-96.4% with stained mode,95.2%-96.5% with NBI
Zachariah et al.(2020) (104)	Retrospective	CADx	5378 images	634 polyp images	Accuracy:94%,NPV:97%
Rodrigues et al.(2021) (105)	Retrospective	CADx	745 images	520 images	Sensitivity:96.0%, Specificity:84%,NPV:91%

## Computer-aided detection system

CADe systems have been developed to increase ADR and adenomas by providing real-time visual information on previously unrecognized polyps (91). Being a standardized second observer, the system can help avoid any missed diagnoses of visible lesions that briefly appear in the field of vision (106) and has been proven to increase polyp detection with high accuracy and consistency (107–109).

Misawa et al. (88) developed an original CNN-based CADe using 411 colonoscopy videos with a sensitivity and specificity of 90% and 63%, respectively, for 50 polyp and 85 non-polyp videos. This compensates for the shortcomings of static images and insufficient samples in previous systems. A subsequent study reported the first real-time application of CNN-based CADe to identify and locate polyps by Urban et al. (89), which showed 96% cross-validation accuracy and an AUC of 0.991 on 8641 images. Notably, four expert reviewers identified 17 additional polyps with CNN compared with eight additional polyps without assistance. Another real-time CAD system CAD EYE trained with linked color imaging technology focused on sessile serrated lesions and achieved the detection rate of 100% (90). The first prospective, randomized controlled trial was conducted by Wang et al. (91) to investigate its effect on ADR. The CADe system significantly increased the mean number of adenomas per patient (0.53 vs. 0.31, p<0.001) and ADR (29.1% vs. 20.3%, p<0.001) than standard colonoscopy, which was mainly attributed to the detection of a greater number of small polyps. In another prospective randomized controlled trial, Su et al. (92) designed a CADe that was able to not only detect colorectal polyps but also measure withdrawal time to improve the performance of endoscopists. The CAD-assisted group had a significantly higher ADR (29% vs. 17%, p < 0.001), a prolonged exposure time (7.03 minutes vs 5.68 minutes, P <0.001), and adequate bowel preparation(87.34% vs 80.63%, *P* =0.023).Yamada et al. (93) developed a system using a supervised DNN and validated it using a dataset of 705 still images of 752 lesions and 4135 still images of noncancerous tissue. The system achieved sensitivity and specificity of 97.3% and 99.0%, respectively, and speed of 21.9 ms/image on average. To overcome the operational bias in previous non-blinded trial, Wang et al. (94) used a double-blind design to evaluate the effectiveness of the system. The ADR was significantly greater in the CADe group than in the sham group, with 165 of 484 patients(34%) versus 132 of 478 patients(28%) having an adenoma detected.

Repici et al. (95) performed a multicenter, randomized trial to evaluate the safety and efficacy of CADe, known as GI Genius from Medtronic. With 685 subjects randomly assigned (1:1), the ADR was higher in the CADe group (54.8%) than in the control group (40.4%) without increasing withdrawal time. In a later study, a random trial was performed with colonoscopists (96). When the data from the above two studies were merged, the application of CADe and colonoscopy indication were correlated with the ADR, and experience seemed to play a secondary role. Because the AMR obtained from tandem colonoscopy is a better indicator than the ADR of how endoscopists performed, Wang et al. (97) compared the specific AMR of CADe colonoscopy with that of routine white-light colonoscopy. The overall AMR was lower with CADe colonoscopy (13.89% vs. 40.00%, P<0.0001), which mean that routine CADe may reduce the incidence of interval CRC. A prospective study conducted by Ishiyama et al. including 1836 patients (98) showed the ADR was higher in the CADe group than in the control group (26.4% vs. 19.9%, OR, 1.32; 95%CI, 1.12–1.57), but there was no significant increase in the advanced neoplasia detection rate (3.7% vs. 2.9%). Another multicenter prospective study firstly took user friendliness into account and presented data on the safety and user experience of Discovery system from Pentax (99). In order to yield better representations of polyp lesions, CADDIE system constructed with novel hybrid 2D/3D network can realize training with smaller static images (110). After testing on 95 videos and 1833 polyp images, it achieved an improvement across all performance metrics included temporal consistency, showing better generalization performance and increased suitability for clinical application.

## Computer-aided diagnosis system

With continuous improvement of the quality of endoscopic imaging systems, optical diagnosis is increasingly applied to the histological prediction of colorectal polyps. However, tissue biopsy remains the gold standard. The accuracy of AI for optical biopsy depends on the extent to which the surface structure reflects the histological characteristics of the lesion (91). CADx can analyze endoscopic images to make a qualitative diagnosis of colorectal tumors with low inter-observer variation. Generally, CAD for colonoscopy is designed to extract features from colonoscopy images or videos and the output includes predicted polyp location or pathology (111). The optical prediction of polyp histology helps guide subsequent treatment and is the key to the “resect and discard” and “detect and leave” strategy (112, 113). Previous studies have demonstrated that en bloc R0 ER of selected colorectal neoplasms confined to the mucosa or superficial submucosa (T1a, with <1000 μm of submucosal invasion and favorable histological features) may be considered a curative resection (114, 115). Therefore, the estimation of the invasion depth is of utmost importance for establishing treatment strategies for colorectal neoplasms (116). The diagnostic capability of the CAD system was evaluated by observing between invasive and less invasive lesions.

Kominami et al. (117) evaluated whether the real-time image recognition system could predict the histological diagnosis of colorectal lesions depicted on NBI. The concordance between the endoscopic diagnosis and CADx output was 97.5%(115/118). Owing to the need for the manual design of imaging features in support vector classifiers, subsequent models are mostly based on AI, especially DL. Similarly, Chen et al. (100) used a CAD model with deep neural network to predict the histopathology of 284 diminutive polyps diagnosed with NBI and achieved an NPV of 91.5% for adenomas. Byrne et al. (101) further demonstrated that the AI model based on DCNN could be used to classify diminutive colorectal polyps. The model achieved 94% accuracy, 98% sensitivity, 97% NPV, and 90% PPV for 106 diminutive polyps. The use of video images can effectively reduce selection bias and simplifies the steps of the clinical work. Kudo et al. (102) performed a retrospective comparative analysis to determine the diagnostic performance of EndoBRAIN, which can identify colon neoplasms by analyzing their microstructures. When the pathological results were used as the standard, the ability of NBI to distinguish neoplastic lesions was significantly higher than or comparable to that of the 30 endoscopists. If high-quality images are available, the system will be a powerful tool for endoscopists with quick response and reproducibility.

To distinguish between invasive cancer and adenomas, Takeda et al. (118) evaluated endoscopic CAD for the diagnosis of invasive CRC. They trained on 5843 images and tested on 200 images with specificity of 98.9% and accuracy of 94.1%. Another CAD system based on ME-NBI was further applied to classify hyperplastic polyps, adenoma/adenocarcinoma lesions, and deep sub-mucosal lesions (119). A single-center, large-scale prospective study (103) showed that endocytoscopy with CADx had an NPV for diminutive rectosigmoid adenomas of 93.7%–96.4% with the stained mode and 95.2%–96.5% with NBI. Considering the missing data, their model met the threshold of 90% recommended by ASGE PIVI. Instead of endoscopy, Zacharia et al. (104) created a CNN-based DL algorithm for real-time *in situ* diagnosis of colorectal polyps. With 5-fold cross validation, the NPV was 97% for diminutive polyps and the surveillance interval concordance achieved 94%. A novel CADx model capable of delineating polyp boundaries and providing localized histological predictions has been presented (105). The histology map increased the transparency and interpretability of the results, and the model was tested on 254 polyps with sensitivity, specificity and NPV of 96%, 84%, and 91%, respectively.

Given the high risk of CRC, to develop a real-time automated polyp detection system can significantly reduce missed diagnosis rates and guide management decisions regarding polyps (120). An ideal CAD system would support the simultaneous detection and classification of polyps to achieve optimal CRC prevention and treatment. In a previous study that evaluated real-time CADx with CADe, the model failed to generate sufficient confidence to predict 15% of the polyps in 125 videos (101). For the remaining 106 polyps, the model achieved an accuracy of 94%, sensitivity of 98%, NPV of 97%, and PPV of 90%. On the one hand, computer analysis of video may reduce differences among endoscopic observers and lead to widespread acceptance of “resect and discard.” On the other hand, AI systems are regarded as low-risk devices that can assist but not replace the work of endoscopists, making it impossible to guarantee the added value of AI in clinical practice (121).

## Limitations and future direction

In recent years, artificial intelligence (AI) has made remarkable progress in medical image recognition and has shown promise in the diagnosis and treatment of gastrointestinal tumors. Medical and engineering institutions are actively conducting a great deal of researches. However, it is critical to overcome the following limitations before it become part of the clinical workflow.

Firstly, most current studies rely on retrospective datasets, especially validation sets, which may be affected by selection bias. On the one hand, due to selection bias, the results obtained in retrospective studies are often better than those obtained in clinical practice. However, since low-quality endoscopic images are often excluded from retrospective studies, they cannot usually determine how to manage low-quality images in clinical practice. To overcome this limitation, multi-center prospective studies, which are necessary for clinical validation, should be prioritized. Secondly, false-positive or false-negative results can be found in some models. The main reason may be the limited quantity and quality of learning materials, which limit the clinical applicability. Therefore, further accumulation of various endoscopic images could reduce these inaccurate results. Video images can be used as learning materials (88, 101)to realize the real-time diagnosis of lesions, and the number and type of images can be further increased. Thirdly, the results lack external validity. Due to differences in genetics, diet, and lifestyle between Chinese and Western populations, the results from one region may not be generalizable to parts of the world with different incidence rates, so adaptability and effectiveness in other areas need to be further explored. In this regard, multicenter studies have been widely conducted in other areas of medicine to evaluate DL systems. Similarly, specific colonoscopy devices were used in most studies, and the adaptability of the model to equipment manufactured by other companies should be further explored to ensure the same performance level. Finally, to date, there is no effective method to verify the status of tumor resection; therefore, future efforts may facilitate AI utilization to distinguish between normal mucosa, adenoma, and submucosal tissue, enabling endoscopists to evaluate resection status (122).

## Discussion

This study reviewed the research and development of AI for gastrointestinal tumors endoscopy. Owing to the insidious clinical symptoms of early gastrointestinal tumors and large variations among endoscopists, the use of AI for lesion detection is not influenced by factors known to influence the size and shape of human observers. AI-assisted systems have evolved from traditional ML algorithms to DL based on neural networks, from still image analysis to real-time video processing. More importantly, it can promote the development of telemedicine since the system is automated and online. With the processing power and high performance of algorithms such as DL, the use of a new era of AI-based assisted endoscopy systems can help endoscopists perform basic tasks such as the early detection and classification of gastrointestinal tumors, and more development and validation is undergoing. Most previous diagnosis process are difficult to be understood by humans, known as “black box”, so it is very necessary to further explore the construction of AI endoscopic systems with man–machine interaction capability. In the training process, a sufficient number of training datasets is essential, and in-depth analysis of as many variables as possible should be carried out. In the validation process, the risk of overtreatment should be taken into account if the specificity is reduced. The selection of included variables may be inappropriate when the sensitivity is reduced. The objectivity and reproducibility of AI technology will enable its further application in the treatment of gastrointestinal tumors, including early detection, pathological identification, risk assessment, treatment guidance, and outcome prediction. AI will likely be introduced into the composition of endoscopic equipment for diagnosis and treatment to improve clinical outcomes. More and more patients and physicians will benefit from the progress of endoscopic AI-assisted systems.

## Author contributions

YX, XLiu, and BL reviewed literature and originally drafted the manuscript. QZ and TM contributed to editing and embellished the manuscript. XLi approved the final version of the manuscript. All authors contributed to the article and approved the submitted version.
